# Providing Ancillary Care in Clinical Research: A Case of Diffuse Large B-Cell Lymphoma during a Malaria Vaccine Trial in Equatorial Guinea

**DOI:** 10.4269/ajtmh.20-1178

**Published:** 2020-11-23

**Authors:** Stephen R. Manock, Ali Mtoro, Vicente Urbano Nsue Ndong, Ally Olotu, Mwajuma Chemba, Antonio E. Sama Roca, Esther Eburi, Guillermo A. García, Carlos Cortes Falla, Julie Niemczura de Carvalho, Jaime Contreras, Baltasar Saturno, Juan de Dios Riocalo, José Luis Nze Mba, Rima Koka, Seung Tae Lee, Hari Menon, L. W. Preston Church, Mitoha Ondo’o Ayekaba, Peter F. Billingsley, Salim Abdulla, Thomas L. Richie, Stephen L. Hoffman

**Affiliations:** 1 Sanaria, Inc., Rockville, Maryland;; 2 Department of Family Medicine, John Peter Smith Hospital, Fort Worth, Texas;; 3 Ifakara Health Institute, Bagamoyo, Tanzania;; 4 Ministry of Health and Social Welfare, Malabo, Equatorial Guinea;; 5 KEMRI Wellcome Trust Research Programme, Kilifi, Kenya;; 6 Medical Care Development International, Malabo, Equatorial Guinea;; 7 Medical Care Development International, Silver Spring, Maryland;; 8 La Paz Medical Center, Malabo, Equatorial Guinea;; 9 Policlínico Dr. Loeri Comba, Instituto de Seguridad Social, Malabo, Equatorial Guinea;; 10 University of Maryland Medical Center, Baltimore, Maryland;; 11 Cytecare Cancer Hospital, Bengaluru, Karnataka, India

## Abstract

Providing medical care for participants in clinical trials in resource-limited settings can be challenging and costly. Evaluation and treatment of a young man who developed cervical lymphadenopathy during a malaria vaccine trial in Equatorial Guinea required concerted efforts of a multinational, multidisciplinary team. Once a diagnosis of diffuse large B-cell lymphoma was made, the patient was taken to India to receive immunochemotherapy. This case demonstrates how high-quality medical care was provided for a serious illness that occurred during a trial that was conducted in a setting in which positron emission tomography for diagnostic staging, an oncologist for supervision of treatment, and an optimal therapeutic intervention were not available. Clinical researchers should anticipate the occurrence of medical conditions among study subjects, clearly delineate the extent to which health care will be provided, and set aside funds commensurate with those commitments.

## INTRODUCTION

Addressing health conditions that arise during clinical trials in places with less than comprehensive medical systems can be difficult. Debate exists about whether and to what extent researchers have obligations to provide *ancillary care* to study participants, that is, medical care that is needed but not necessary to prevent or mitigate harm caused by participation in research, or answer questions that are the focus of the study.^1–4^ The evaluation and treatment of serious illnesses can incur a substantial burden in terms of researchers’ time and funds.^2^

We report a case of diffuse large B-cell lymphoma that was diagnosed during a double-blind, placebo-controlled trial of whole sporozoite candidate malaria vaccines PfSPZ Vaccine^5–10^ and PfSPZ-CVac^11^ in Equatorial Guinea (ClinicalTrials.gov number NCT02859350). The study was approved by the Comité Ético Nacional de Guinea Equatorial, additionally reviewed by the MaGil Institutional Review Board (IRB) in Rockville, MD, the Ifakara Health Institute IRB in Tanzania, and the Ethics Committee of Northwestern and Central Switzerland (EKNZ), and was conducted under a U.S. Food and Drug Administration Investigational New Drug application. All study participants underwent an informed consent process, had a thorough medical history, physical examination and laboratory screening, and were deemed healthy before being enrolled. The study protocol specified that participants would receive general outpatient healthcare services from Equatoguinean study physicians at the research center during the course of the trial. In case of medical conditions that required more specialized or inpatient care, a referral would be made to a tertiary care hospital near the research center (La Paz Medical Center). If the cost of treatment for an illness unrelated to study participation exceeded the amount budgeted for medical care by the trial, the participant would be responsible for obtaining this care through the Ministry of Health and Social Welfare of Equatorial Guinea. Provision of health care beyond the scope of services provided in the country was not anticipated in the study protocol or the informed consent form, and the ethics committees did not provide specific guidelines on the topic.

## CASE REPORT

**Figure 1.**
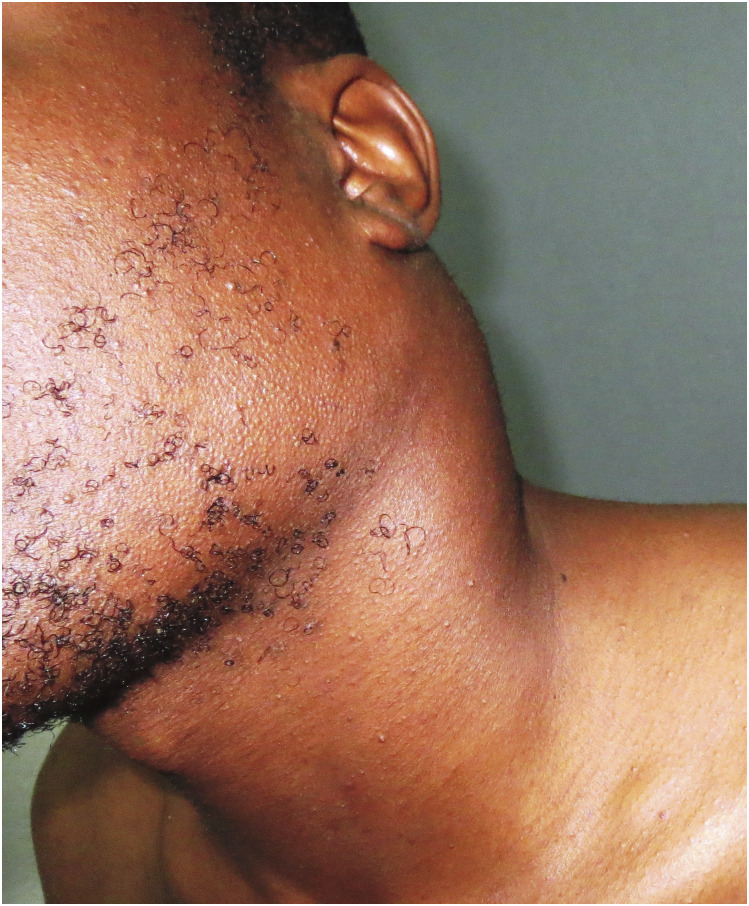
Subject with prominent, progressive cervical lymphadenopathy following treatment for suspected bacterial lymphadenitis, tuberculous lymphadenitis, and toxoplasmosis. This figure appears in color at www.ajtmh.org.

A previously healthy young adult male subject developed tender left posterior cervical lymph nodes 3 weeks after administration of his first dose of investigational product (later determined to be normal saline placebo). The initial diagnosis was acute bacterial lymphadenitis, and treatment with oral amoxicillin and ibuprofen was given by a study physician. When the lymphadenopathy slowly progressed over the next 2 months, a referral was made to an internist at La Paz Medical Center. There the patient was found to have eosinophilia, a positive toxoplasmosis IgG titer, and mild cervical, supraclavicular, axillary, mesenteric and mediastinal lymphadenopathy by computerized tomography (CT). The patient was referred to the national tuberculosis (TB) program for empiric treatment of suspected TB lymphadenitis. Oral isoniazid, rifampin, pyrazinamide, and ethambutol were given for 4 weeks, during which time the cervical lymph nodes increased in size. Oral trimethoprim–sulfamethoxazole and clindamycin were then given for 10 days for possible toxoplasmosis, but following treatment, the left cervical lymph nodes had enlarged to 10 cm in diameter (see Figure 1), and cervical lymph nodes were now also palpable on the right. The patient complained of left-sided neck pain that radiated down the left arm, throat discomfort with swallowing, and feeling feverish at night. There had been a weight loss of 3 kg over the preceding 2 months, but no sweats or impairment of breathing. A lymph node biopsy was performed at La Paz Medical Center and sent to Policlínico Dr. Loeri Comba in Malabo, Equatorial Guinea, where the pathologist diagnosed a non-Hodgkin lymphoma. Biopsy material was hand-carried to the University of Maryland Medical Center where further testing narrowed the diagnosis to diffuse large B-cell lymphoma. The study team had determined that there were no oncologists and no options for immunochemotherapy in Equatorial Guinea and were concerned that the patient would likely die without treatment. After a search for potential treatment centers, it was established that the Cytecare Cancer Hospital in Bangalore, India, was well-equipped to provide high-quality care for the patient. Oncologists at the University of Maryland and Cytecare agreed that the best treatment would be six cycles of rituximab, cyclophosphamide, doxorubicin, vincristine, and prednisone (R-CHOP). Eleven days after his diagnosis was made, the patient arrived in Bangalore accompanied by his cousin and the research study’s head nurse. The nurse remained with the patient during his first 10 days of evaluation and treatment. Full-body positron emission tomography (PET) showed diffuse hypermetabolic activity and lymphadenopathy. Lactate dehydrogenase was elevated, but there was no anemia and no evidence of extranodal involvement. The patient was determined to have stage IIIBx disease with a Revised International Prognostic Index of 2, predictive of a 4-year overall survival rate of 79% with treatment.^12^ For the next 4 months, the patient lived in Bangalore with his cousin. He received six cycles of R-CHOP as an outpatient with only mild side effects, and there was a rapid reduction in the size of his cervical lymph nodes. A posttreatment PET scan showed resolution or regression of all previously observed lymphadenopathy and a complete metabolic response to immunochemotherapy, but some new right hilar lymphadenopathy and a small right pleural effusion were seen. Thoracentesis was performed. The pleural fluid was exudative and showed numerous leukocytes (primarily lymphocytes), with no bacteria seen on Gram stain, a negative smear for acid-fast bacilli (AFB), and a negative bacterial culture. Cytopathology carried out on the pleural fluid showed predominantly lymphoid cells which expressed CD3, but not CD20 or CD79a, markers, which was felt to be inconsistent with malignancy. Five days later, the right pleural effusion had increased in size, prompting repeat thoracentesis. Pleural fluid adenosine deaminase activity was elevated, but GeneXpert and a repeat AFB smear were negative. The presumptive diagnosis was pleural TB, possibly secondary to reactivation of previously unrecognized latent TB post-immunochemotherapy. Treatment was begun with oral isoniazid, rifampin, pyrazinamide, and ethambutol. A follow-up ultrasound performed after 2 weeks of therapy showed near resolution of the pleural effusion, supporting the diagnosis of TB. The patient traveled home to Equatorial Guinea 2 days later, where he was promptly referred to the national TB program to complete 6 months of standard directly observed therapy. Physical examination by a physician was advised every 3 months for surveillance, with ultrasound or CT evaluation of any clinical recurrence of cervical lymphadenopathy, or symptoms suggesting intra-abdominal lymphadenopathy. Given his excellent clinical and radiological response to therapy, he was estimated to have a 50–60% likelihood of complete cure.^13^ The total cost of evaluation and treatment of this patient at Cytecare was 10,521 U.S. dollars, plus an additional 36,128 U.S. dollars for airfare, travel documents, housing, and living expenses in India for the patient, his cousin, and the study head nurse. It was agreed, in writing, that the cost of any future medical care would be the responsibility of the patient and his family. As of 32 months after his final dose of immunochemotherapy, the patient was free of clinical signs and symptoms of lymphoma.

## DISCUSSION

Addressing medical needs that arise during the course of clinical research can be challenging, particularly in resource-limited settings which lack state-of-the-art healthcare services for serious conditions that arise during the course of the trial but are not related to trial participation. There has been considerable debate about whether researchers have an ethical duty to provide care to study subjects, particularly for conditions that are unrelated to their participation in research.^1,4,14–16^ Some have argued that providing ancillary medical care has the potential to compromise and overwhelm research studies,^1,4,15^ and that requiring such care could potentially discourage researchers from working in underserved populations.^4^ Merritt et al.^17^ propose that nonclinical, public health researchers may be less obliged to address health needs if they do not have the needed expertise to address the problem, particularly if the costs of doing so are inordinate, or other organizations are available to meet the need. Others debate whether incidental medical findings must even be reported to study participants, or referrals be made for further evaluation and treatment.^18^ Garrett^15^ optimistically suggests that research protocols be designed to minimize or eliminate the possibility of incidental findings.

Ethical arguments in support of an obligation to provide ancillary care tend to be based on the *duty of rescue*, which holds that everyone has a responsibility to render life-saving assistance to others in need if it is within their power to do so,^1,14,16,19,20^ provided that this does not incur “serious sacrifice or risk”^1^ or “conflict with some weighty moral aim.”^16^ Beyond that general duty, it has been suggested that by granting permission to study their body and its functions, subjects “effectively entrust the researchers with special responsibilities to look after the needs they discover.”^21^ Ancillary medical care has been called “morally obligatory,”^22^ and “an integral and necessary part of ethical research with human beings,” particularly if conditions are severe, acute, and/or would have serious consequences if left unmet.^3^ It has been proposed that an increased responsibility to provide care exists in situations where researchers have a long-term, professional relationship with study participants,^3,4^ where doing so is within the expertise of the team,^4^ or when researchers are “in a unique position to help participants.”^2^ Some have suggested that the duty to address health needs must be anticipated during the planning of research studies, and funds specifically budgeted to provide ancillary care.^1,2^ In the case of the current study, 166,373 U.S. dollars was allotted as self-insurance for healthcare expenditure for all 135 trial participants, although this did not include a provision for sending patients and staff overseas. Obtaining commercial medical insurance for each research subject would have been another option, although in most cases, this would be prohibitively expensive. In the end, a total of 120,308 U.S. dollars was spent on medical care for all subjects in this study, notwithstanding the unexpected costs of treatment abroad for this patient with lymphoma.

There is disagreement among those who hold the position that researchers have an obligation to address the health needs of study participants as to the extent of ancillary care that is required. Dickert and Wendler^4^ propose potential levels of ancillary care: providing diagnostic information, making referrals for care, providing treatment, or paying for treatment. They note that obligations to provide care may be limited by the researchers’ level of expertise or if the care is prohibitively expensive. It has been argued that researchers do not necessarily have a responsibility to provide fully comprehensive medical care to study participants.^2^ Furthermore, debate exists as to whether researchers from high-income countries should be required to provide the same level of care that is available in their home countries, or whether “it is ethically acceptable to provide treatments based on what is routinely available in the host country.”^23^ Benatar and Singer have argued that although providing the same standard of care in resource-limited countries as in the industrialized world may not be realistic, the goal should be to provide the highest achievable level of care.^24^ They propose that this should include providing subjects with treatment that would not ordinarily be available to them in the country where the trial is being carried out. Establishing strict rules as to what level of ancillary care is universally required of researchers working in resource-limited settings has proven to be exceedingly difficult.^4,16^

In the current case, the provision of in-country ancillary care for study participants was anticipated and budgeted. The study protocol established that comprehensive primary care would be provided free of charge by physicians at the research center. Healthcare needs that could not be addressed by study physicians were to be referred for appropriate specialty care, and this care was also to be paid for by the trial. Treatment for conditions unrelated to trial participation that exceeded the medical care budget of the study was to have been sought through the Ministry of Health and Social Welfare. However, after months of professional interaction with this participant, study physicians were faced with the challenge of securing potentially life-saving treatment for his lymphoma in a country without PET scanning for diagnostic staging, an oncologist, or the availability of immunochemotherapy. The need for medical care outside the country had not been specifically foreseen, but there were funds set aside for medical emergencies as stated previously. The mean cost of the initial 5 months of treatment for patients with diffuse large B-cell lymphoma in the United States was estimated to be 72,010 U.S. dollars,^25^ plus an estimated 56,500 U.S. dollars for airfare, travel documents, lodging, and living expenses in the Washington, D.C. area.^26^ This is nearly three times the total expense of being treated in Bangalore and would have been cost-prohibitive. Thus, being able to obtain reasonably priced, high-quality care in India was critical in this case. India is a well-known destination for medical care and has an established infrastructure to receive large numbers of foreign patients,^27–29^ with a sizable proportion traveling there from Africa.^30^ Although the cost of care, travel, and an extended stay in India was not anticipated, they could be accommodated within the budget of a properly funded research program.

Beyond financial costs, it should be recognized that the successful evaluation and treatment of this study participant required a large investment of time and effort by a multinational, multidisciplinary team. Local medical resources were used to the fullest extent possible and included primary care, evaluation at a tertiary care hospital, surgical referral for biopsy, pathology consultation, and TB care. Remote consultations with oncology, pathology, and infectious disease specialists in the United States occurred during his clinical course. Considerable administrative support was required to secure passports and visas, and arrange for overseas travel, lodging in India, provision of per diems, and payment of hospital bills. Our head nurse’s presence in India during the initial days of treatment was instrumental in transitioning care to the oncologist there. Several study team members—administrators, community outreach workers, nurses, and physicians—maintained ongoing communication with the patient during his stay in India, to encourage him and ensure that his needs were being met. Finally, staff at Cytecare Cancer Hospital in Bangalore provided excellent oncology care and a thorough diagnostic workup for the pleural effusion that developed late in the patient’s course, all the while fielding inquiries from the study sponsor in the United States and the research team in Equatorial Guinea.

In addition to ethical and humanitarian considerations, securing definitive medical care for this lymphoma patient had pragmatic benefits for the malaria vaccine research program. This study was part of a series of clinical trials financed by the government of Equatorial Guinea and international oil and gas companies working in the country. Providing high-quality medical care beyond what was required by the study protocol and consent form was viewed positively by all stakeholders. So rather than detracting from research, this relatively large expenditure on the health of one study participant may serve to further the goals of the overall research program. Others have noted that providing ancillary care is a wise investment when researchers intend to conduct a series of studies with the same population.^3^

There are potential pitfalls to providing the sort of comprehensive, specialized care that this lymphoma patient received. Offering free medical care could serve as an inducement to participate in a clinical trial, particularly in settings where health care is difficult to access.^3,23^ Such an inducement could tempt potential subjects to conceal significant medical conditions at the time of study enrollment to improve their access to otherwise costly care. Also, the provision of comprehensive health care to study participants, and not to others in the community who may have limited access, might run counter to the ethical principal of justice. Perhaps, more equitable approaches that would improve care for the larger population, and not just study subjects, should be sought.^15^ Finally, there is a concern that providing this level of health care to some individuals in a clinical trial could distract the attention of researchers, thereby compromising the quality of data collection or the safety of other study participants.

In summary, this is an example of excellent medical care being provided for a study participant who developed a life-threatening illness during a clinical trial in a setting that lacked the healthcare services required for provision of optimal medical treatment for his condition. This case highlights the importance of clinical investigators anticipating such occurrences and deciding before initiation of the trial what their responses to such illnesses will be. Regardless of what the final decision is, the plan should be clearly communicated in the informed consent form, the protocol, and clinical trial agreements. If the decision is made to provide ancillary care, funds must be set aside in advance for its support.
